# Fluoroquinolones for TB preventive treatment of contacts exposed to persons with multidrug-resistant TB: a systematic review and meta-analysis

**DOI:** 10.5588/ijtldopen.26.0019

**Published:** 2026-06-15

**Authors:** H. Sidhu, Y. Hamada, S. Carroll, D. Falzon, A. Kanchar, K. Schenkel, D. Menzies

**Affiliations:** 1 McGill International TB Centre, McGill University, Montreal, QC, Canada;; 2 Research Institute McGill University Health Centre, McGill University, Montreal, QC, Canada;; 3 Institute for Global Health, University College London, London, UK;; 4 Department of Epidemiology, Biostatistics and Occupational Health, McGill University, Montreal, QC, Canada;; 5 Global TB Programme, World Health Organization (WHO), Geneva, Switzerland;; 6 Health Emergency Intelligence and Surveillance Systems (WSE), Health Emergencies Programme, WHO, Geneva, Switzerland.

**Keywords:** tuberculosis, TBI, TPT, drug-resistant TB, MDR-/RR-TB

## Abstract

**BACKGROUND:**

We evaluated the safety, acceptability, and cost-effectiveness of all published studies on fluoroquinolone (FQ)-based TB preventive treatment (TPT) regimens in all settings and populations.

**METHODS:**

We searched for studies published between February 2014 and September 2023 using PubMed, Embase, Turning Research Into Practice (TRIP), Global Health Library, and the Cochrane Central Register of Controlled Trials. Meta-analysis was conducted using random effects models.

**RESULTS:**

Twenty-two observational and modelling studies evaluating FQ-based TPT were included, assessing outcomes of safety (n = 16), acceptability (n = 9), and cost-effectiveness (n = 2). Only 2% (95% confidence interval [CI]: 0%–4%) of children and adolescents and 8% (95% CI: 2%–14%) of adults discontinued FQ monotherapy due to drug-related adverse events (AEs). Compared to FQ monotherapy, rates of drug-related AEs of any severity were non-significantly higher with multidrug FQ regimens without pyrazinamide (PZA). Pooled discontinuation rates were 50% (95% CI: 34%–67%) among children and 57% (95% CI: 16%–98%) among adult contacts treated with PZA-containing FQ-based regimens. Pooled acceptance rates exceeded 80% among all groups. FQ-based TPT was cost-effective to avert TB-related deaths and loss of quality-adjusted life-years.

**CONCLUSION:**

These results complement the evidence from two recent randomised trials. FQ monotherapy is a safe, acceptable, and cost-effective TPT regimen for contacts exposed to multidrug-resistant or rifampicin-resistant TB disease.

Drug-resistant TB (DR-TB) continues to be a threat to public health. In 2023, there were an estimated 400,000 persons with multidrug-resistant or rifampicin-resistant TB (MDR-/RR-TB).^1^ MDR-TB and RR-TB are of particular concern since treatment is much more complex than for drug-sensitive TB, with greater costs and toxicity, and reduced effectiveness.^2^ Providing TB preventive treatment (TPT) to persons at high-risk of developing disease is a critical component of the End TB strategy.^3^ Although multiple randomised trials have demonstrated efficacy and effectiveness of several TPT regimens in contacts exposed to drug-susceptible TB, there is little evidence of the efficacy and safety of TPT for persons with exposure to patients with MDR-/RR-TB disease. Two randomised placebo-controlled trials were recently published that evaluated levofloxacin (LFX) TPT for household contacts of MDR-TB patients among adults (primarily) in Vietnam (VQUIN)^4^ and children in South Africa (TB-CHAMP).^5^ These compared the incidence of bacteriologically confirmed TB in those taking LFX compared to placebo as the primary objective, with safety, cost-effectiveness, and acceptability as secondary outcomes. While these trials and a meta-analysis of the two trials combined^6^ provided high-quality evidence of safety and effectiveness, there remains a need for evidence of safety and acceptability of fluoroquinolone (FQ)-based TPT from a wider range of settings and populations. A systematic review and meta-analysis conducted in 2017 reported efficacy, safety, and completion of TPT for contacts of infectious MDR-TB patients.^7^ A more recent review published in 2024^8^ reported on effectiveness, safety, and cost-effectiveness of TPT for contacts of DR-TB.

In addition to updating evidence on these outcomes, we assessed evidence of acceptability, feasibility, and impact on equity of FQ-based TPT among persons exposed to patients with MDR-/RR-TB, to inform a WHO Guideline Development Group (GDG) meeting in December 2023.

## METHODS

This article was an update to a previous unpublished review on TPT for contacts of MDR-TB conducted in 2014 for the development of 2015 WHO guidelines.^9^ Two separate searches were conducted using the same search strategy as the 2014 review in PubMed, Embase, Turning Research Into Practice (TRIP), and Global Health Library. For randomised trials, the Cochrane Central Register of Controlled Trials (CENTRAL) was also searched. Detailed search terms can be found in Supplementary Data. The first search was limited to studies published from 1 February 2014 to 29 November 2016 in preparation for a GDG meeting in May 2017. The second search was limited to studies published from 1 June 2016 to 13 September 2023, in preparation for the GDG meeting in December 2023. There were no language restrictions for any of the searches. Relevant studies were also identified from the reference lists of selected studies. These searches were merged, and full texts selected from both searches were reviewed to provide a full review of evidence on FQ-based TPT.

We included randomised controlled trials (RCTs) and observational studies, including cohort studies, case-control studies, case series, cross-sectional qualitative studies, and population-based studies. All definitions of contacts (i.e., close, casual, and household) exposed to pulmonary MDR-TB disease were accepted. Included studies reported at least one of the following outcomes: treatment-related adverse events (AEs), treatment discontinuation due to AEs, TPT completion, emergence of additional FQ resistance in TB strains or in microbiome other than TB strains, resources required for implementation, impact on equity, patient and health care worker values and acceptability, and feasibility of FQ preventive treatment. Modelling studies were included if they reported cost-effectiveness. Literature reviews, abstracts, case reports, opinion articles, and grey literature were not included.

Studies measuring acceptability were split into three sub-outcomes for this review: acceptance, willingness, and acceptability of FQ-based TPT regimens. Acceptance was defined as the proportion of eligible contacts, or caregivers of eligible contacts, who started TPT when offered. Willingness was defined as the proportion of contacts and caregivers of child contacts who stated they were open to taking (or providing to their children) a hypothetical, newly developed MDR TPT regimen. Finally, acceptability was defined as the ability to adhere to the TPT regimen as directed.

### Selection of publications, data extraction, and quality assessment

Two reviewers (either YH and KS, or HS and SC) independently reviewed all titles and abstracts to identify potentially eligible studies. The same reviewers then independently assessed all full-text articles which were considered potentially eligible. Disagreements between reviewers were resolved by consensus and discussions with a third reviewer (HG or DM). The following information was independently extracted from included studies: study characteristics (study design, date, setting, sample size), details of the study population (i.e., demographic characteristics, specific subpopulations if present, and other risk factors), TPT regimen and comparator (if any), treatment outcome(s) as outlined above, and information needed for quality assessment.

For observational studies evaluating the safety of MDR TPT, we used items from the Newcastle–Ottawa Scale^10^ to determine risk of bias (see Supplementary Data Table S2). Cross-sectional studies of acceptability were evaluated using the AXIS tool^11^ (Supplementary Data Table S3), and studies on cost-effectiveness were evaluated using the Joanna-Briggs Institute critical appraisal tool for economic evaluations^12^ (Supplementary Data Table S4). Each item of the relevant quality assessment forms was rated as having either a ‘high’, ‘medium’, or ‘low’ risk of bias, before making an overall assessment of study quality. Two reviewers independently evaluated the quality of the included articles and differences were resolved through discussion (until consensus was reached).

### Statistical analysis

Outcomes that were judged to be the same, and were reported in at least three studies, were pooled using random effects models with the inverse variance method to estimate proportions and 95% confidence intervals (CIs). To address clinical heterogeneity, meta-analyses were stratified by age group (children/adolescents or adults) and TPT regimen type (FQ monotherapy, FQ-based multidrug regimen without pyrazinamide [PZA], FQ-based multidrug regimen with PZA). Sensitivity analyses excluding cohorts with 10 or fewer participants were performed to examine robustness of pooled estimates. All meta-analyses were performed using the ‘meta’ package on RStudio version 2024.04.1+748.

## RESULTS

**Figure 1.**
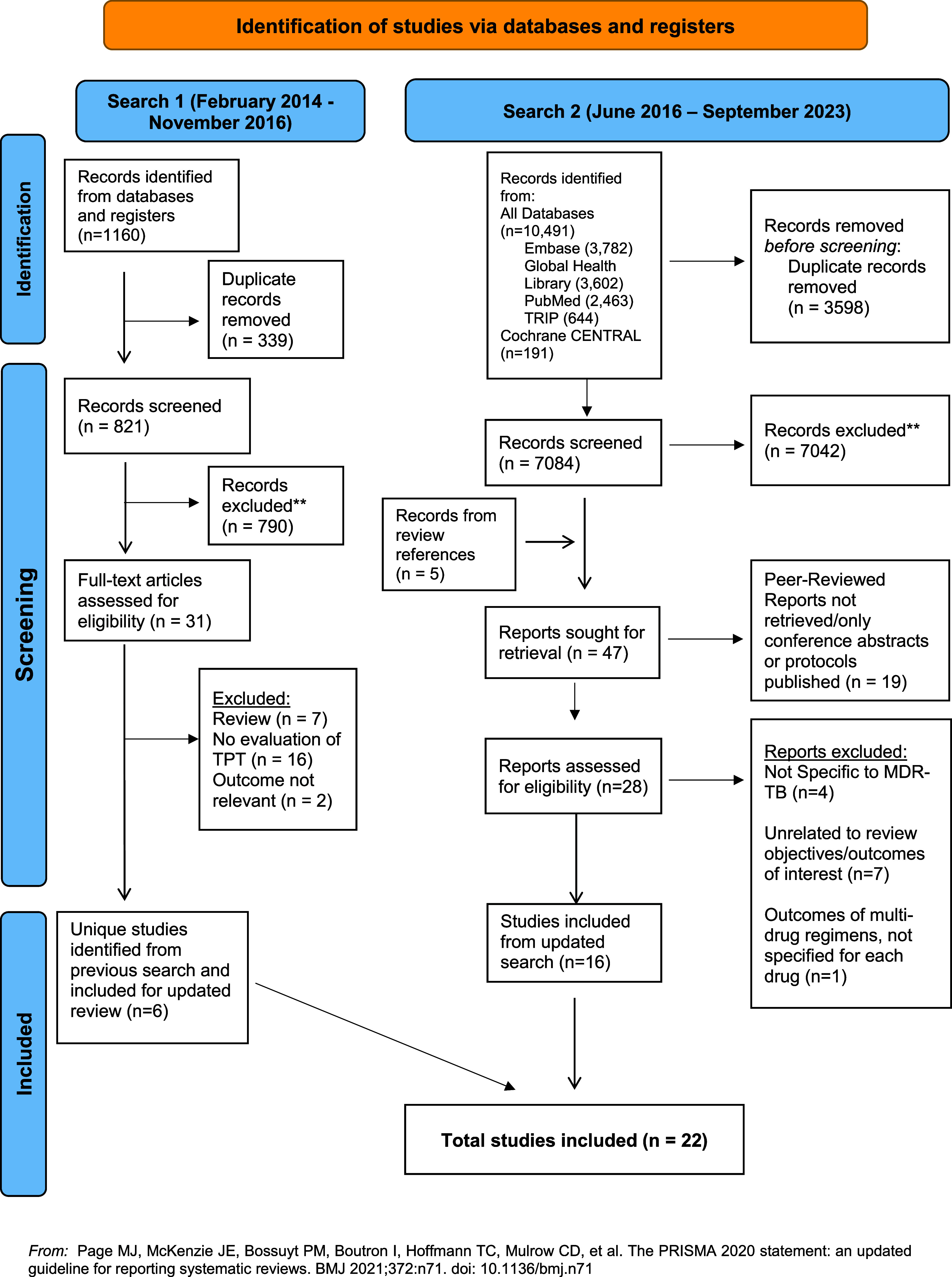
PRISMA flow diagram summarising the screening process. TRIP = Turning Research Into Practice; TPT = TB preventive treatment; MDR-TB = multidrug-resistant TB.

From a total of 7,905 unique articles identified from databases and additional references, 22 studies met our inclusion criteria (Figure 1). Characteristics of included studies are summarised in Supplementary Data Table S1. No RCTs were found. We did not find any studies evaluating the impact of FQ-based TPT on equity.

Sixteen observational studies evaluated the safety of FQ-based TPT given to contacts of persons with MDR-TB disease, of which 12 were among children and adolescents and four were among adults (Table 1). Nine studies reported on the acceptability of FQ-based TPT; eight of these reported quantitative measures, and one was a qualitative study of acceptability of a novel paediatric LFX formulation among caregiver and child contact pairs (Table 2). Two studies evaluated the cost-effectiveness of providing LFX to household contacts: one considered household contacts in the USA and the other the global population of household contacts under the age of 15 (Table 3).

**Table 1.** Summary of included studies evaluating the safety (drug-related adverse events and completion/discontinuation rate) of preventive fluoroquinolone monotherapy or fluoroquinolone-based TPT among contacts exposed to MDR-/RR-TB.

Author (year)	Setting	Population (size and age)	TPT regimen	Outcome definition/type	Outcome estimates
Studies including children and adolescent contacts exposed to MDR-/RR-TB					
Adler-Shohet et al.^13^	School Classroom in California, USA	Children in close contact with a person with MDR-TB disease, with a positive TST (n = 31) (mean age = 9.6 years, range 6–13)	LFX + PZA (n = 26)	Drug-related AEs reported	26/26
				Treatment discontinuation due to drug-related AEs	11/26
				Proportion completing any LFX-based TPT	14/26 (54%)
				Proportion completing LFX + PZA	8/26 (31%)
				Proportion switched to LFX monotherapy and completing	6/11 (55%)
Apolisi et al.^14^	Khayelitsha, Cape Town, South Africa	Children and adolescents aged 0–18 who were household contacts of a person with MDR-TB disease (n = 95) (median age = 8.5 years)	LFX (n = 79)	Mild or moderate AEs reported during the course of TPT	LFX: 8/79
			INH (n = 9)	Serious AEs^A^	INH: 0/9
			DLM (n = 7)	Treatment discontinuation due to drug-related AEs	DLM: 4/7
					None
					LFX: 3/79
					INH: 0/9
					DLM: 4/7
Bamrah et al.^15^	Chuuk, Federated States of Micronesia	Household and health care facility contacts (all ages) exposed to persons with MDR-TB disease (n = 119) (median age = 24 years, 6 children <5 years of age, 43 participants <18 years of age)	MFX (n = 46)	Drug-related AEs reported	56/104
			MFX + EMB (n = 24)	Serious AEs^A^	0/104
			LFX (n = 5)	Treatment discontinuation due to drug-related AEs	4/104
			LFX + EMB (n = 17)	Proportion completing…	5/5 (100%)
			LFX + ETH (n = 12)	LFX monotherapy	36/46 (78%)
				MFX monotherapy	93/104 (89%)
				Any TPT	
Catho et al.^16^	Hospices Civils de Lyon, France	Children who were contacts of adults with MDR-TB disease (n = 46)	LFX + PAS (n = 1)	Drug-related AEs reported	0/1
Chang et al.^17^	Chest Clinics, New South Wales, Australia	Contacts (all ages) of persons diagnosed with MDR-TB disease (n = 18)	MFX (n = 7)	Drug-related AEs reported	0/7
				Proportion completing TPT	7/7
				Treatment discontinuation due to drug-related AEs	0/7
Denholm et al.^18^	Victorian Department of Health, Australia	Contacts (all ages) of persons with MDR-TB disease, and diagnosed as having TB infection (n = 49) (median age = 27 years)	MFX (n = 2)	Drug-related AEs reported	4/11
			MFX + EMB (n = 2)	Treatment discontinuation due to drug-related AEs	2/11 (CFX)
			CFX (n = 2)	Proportion completing TPT	9/11
			CFX + PZA (n = 1)		
			RIF + PZA + EMB (n = 1)		
			INH + PZA (n = 1)		
			EMB + PZA (n = 2)		
Garcia-Prats et al.^19^	Day Care in Cape Town, South Africa	Children exposed to persons with MDR-TB disease (n = 34) (median age = 3.9 years)	OFX + EMB + INH (high dose) (n = 24)	Drug-related AEs reported	2/24
				Treatment discontinuation due to drug-related AEs	2/24
				Proportion completing TPT	21/24 (88%)
Garcia-Prats et al.^20^	Cape Town, South Africa	Children <5 years of age who were household contacts of a person with MDR-TB disease (n = 27) (median age of 24 of the 27 children who provided safety data = 2.1 years)	Novel 100 mg paediatric LFX dispersible tablets (n = 27)	Grade 1 or 2 AEs^B^ at least possibly related to LFX	2/27
				Grade 3 or 4 AEs^B^ at least possibly related to LFX	0/27
				Treatment discontinuation due to drug-related AEs	0/27
Gureva et al.^21^	Arkhangelsk Region, Russian Federation	Children aged <18 years who were household contacts of a person with MDR-TB disease (n = 72) (median age = 7 years, 20 children <5 years of age)	OFX (n = 3) or MFX (n = 55)	Grade 1 or 2 drug-related AEs^B^	6/58
				Treatment discontinuation due to drug-related AEs	1/58
				Proportion completing TPT	52/58 (90%)
Malik et al.^22,23^	Karachi, Pakistan	Household contacts (of all ages) exposed to persons with MDR-TB disease (n = 172) (median age = 7 years)	ETH + LFX (n = 54)	Grade 1 or 2 drug-related AEs^B^	ETH + FQ: 20/59
			ETH + MFX (n = 5)	Proportion completing TPT	EMB + FQ: 16/113
			EMB + LFX (n = 102)	Children <5 years old reporting an AE	121/172 (70%)
			EMB + MFX (n = 11)	Children aged 5–19 reporting an AE	6/61 (10%)
				Adults >19 years old reporting an AE	23/92 (25%)
					7/19 (37%)
Ridzon et al.^24^	California, USA	High school students and teachers exposed to a student with infectious pulmonary MDR-TB (n = 22) (median age = 17 years)	OFX + PZA (n = 22)	Drug-related AEs reported	18/22
				Serious AEs^A^	3/22
				Treatment discontinuation due to drug-related AEs	13/22
				Proportion completing TPT	9/22
Seddon et al.^25^	Cape Town, South Africa	Children <5 years of age, or HIV-positive children aged <15, who were contacts of persons with MDR-TB disease (n = 186) (median age = 2.8 years)	OFX + EMB + INH (high dose) (n = 186)	Drug-related AEs reported^C^:	152
				Grade 1 (not mutually exclusive)	40
				Grade 2 (not mutually exclusive)	6/186
				Grade 3	0/186
				Grade 4	0/186
				Treatment discontinuation due to drug-related AEs	
Studies among only adult contacts exposed to MDR-/RR-TB					
Bedini et al.^26^	Penitentiary in Modena, Northern Italy	Adult prison inmates in contact with a person with MDR-TB disease (n = 17) (mean age = 34 years, range 21–59)	LFX + PZA (n = 12)	Drug-related AEs reported	9/12
				Treatment discontinuation due to drug-related AE	7/12
				Proportion completing TPT	5/12 (42%)
Lou et al.^27^	University of Pittsburgh Medical Center Health System, USA	Adult solid organ transplant recipients with possible exposure to a single person with MDR-TB (n = 48) (mean age = 51.3 years, SD = 11.2)	LFX + PZA (n = 48)	Treatment discontinuation due to drug-related AEs	32/48
				Proportion completing TPT	13/48 (27%)
Papastavros et al.^28^	Hamilton, ON, Canada	Case series of adults with positive TST and exposure to person with MDR-TB (n = 17) (median age = 36 years, range 18–58)	LFX + PZA (n = 17)	Drug-related AEs reported	17/17
				Treatment discontinuation due to drug-related AEs	17/17
Trieu et al.^29^	Housing and harm reduction facilities in New York City, USA	HIV-positive, close contacts of persons with MDR-TB disease (n = 241)	MFX (n = 26)	MFX discontinuation due to drug-related AEs	3/26
			MFX + PZA (n = 24)	Proportion completing MFX TPT	16/26 (62%)
				Proportion completing MFX + PZA TPT	14/24 (58%)

AE = adverse event; DLM = delamanid; CFX = ciprofloxacin; EMB = ethambutol; ETH = ethionamide; FQ = fluoroquinolone; INH = isoniazid; LFX = levofloxacin; MDR-TB = multidrug-resistant TB; MFX = moxifloxacin; OFX = ofloxacin; PAS = para-aminosalicylic acid; PZA = pyrazinamide; RIF = rifampicin; TPT = TB preventive treatment; TST = tuberculin skin test.

A In the study by Apolisi et al.,^14^ seriousness of adverse events was determined by the treating clinician. Bamrah et al.,^15^ defined serious AEs as those leading to hospitalization or irreversible morbidity. Finally, Ridzon et al.^24^ did not pre-define serious AEs. In this study, participants were routinely monitored by nurses, and serious AEs in this study were as follows: angioedema in one participant that required emergency hospitalization and significantly elevated liver enzymes with minimal clinical symptoms in two participants (discovered through routine serology).

B Adverse events were classified according to grading tables of the National Institute of Allergy and Infectious Diseases’ Division of AIDS.

C Adverse events were categorised using the National Institute of Allergy and Infectious Diseases’ Division of Microbiology and Infectious Diseases grading system.

**Table 2.** Summary of included studies evaluating acceptability of preventive fluoroquinolone monotherapy, fluoroquinolone-based regimens, and hypothetical preventive treatment medication amongst child, adolescent, and adult household contacts and caregivers.

Author (year)	Setting	Population	TPT regimen	Acceptability outcome	Outcome estimate
Studies evaluating willingness or acceptance to initiate fluoroquinolone-based TPT					
Adler-Shohet et al.^13^	School Classroom in California, USA	Children in close contact with a person with MDR-TB disease, with a positive TST (n = 31) (mean age = 9.6 years, range 6–13)	LFX + PZA	Proportion of parents/caregivers that agreed to child starting TPT	26/31 (83.9%)
Bamrah et al.^15^	Chuuk, Federated States of Micronesia	Household and health care facility contacts (all ages) exposed to a person with MDR-TB disease (n = 119) (median age = 24, 6 children <5 years of age)	MFX	Proportion of contacts and caregivers that agreed to start TPT	104/119 (87%)
			MFX + EMB		
			LFX		
			LFX + EMB		
			LFX + ETH		
Bedini et al.^26^	Penitentiary in Modena, Northern Italy	Adult prison inmates in contact with a person with MDR-TB disease (n = 17) (mean age = 34, range 21–59 years)	LFX + PZA	Proportion of adult contacts that accepted MDR TPT (n = 5)	12/17 (70.6%)
Gureva et al.^21^	Arkhangelsk Region, Russian Federation	Children <18 years old who were household contacts of a person with MDR-TB disease (n = 72) (median age = 7)	OFX or MFX	Proportion of parents/caregivers that agreed for child to be started on TPT with OFX/MFX	58/72 (80.5%)
Malik et al.^22^	Karachi, Pakistan	Household contacts of all ages exposed to persons with MDR-TB disease (n = 215) (median age = 7)	FQ (LFX/MFX) + ETH OR EMB	TPT-eligible participants who accepted treatment	172/215 (80%)
Rouzier et al.^30^	Eight countries – Botswana (1 site), Brazil (1), Haiti (1), India (2), Kenya (1), Peru (2), South Africa (7), and Thailand (1)	Adult and adolescent household contacts exposed to MDR-TB who reported caring for children <13 years or a dependant of any age (n = 299) (median age = 35)	One-time questionnaire: relevant caregiver-level factors evaluated were identified through literature review and informed by the Health Belief Model	Proportion of caregivers willing to… administer daily MDR TPT pill to children have their children complete prerequisite steps to determine MDR TPT eligibility.	278/299 (92.9%)
					283/299 (94.7%)
Suryavanshi et al.^31^	Eight countries – Botswana (1 site), Brazil (1), Haiti (1), India (2), Kenya (1), Peru (2), South Africa (7), and Thailand (1).	Adolescent and adult household contacts (≥13 years of age) of persons with MDR-TB disease (n = 743) (median age = 33)	One-time questionnaire assessing willingness to take hypothetical, newly developed MDR TPT	Percentage of household contacts’ willing to…	79% (site-level median, 90% [IQR, 84%–95%])
				…take hypothetical, newly developed MDR TPT	70% (site-level median, 80% [IQR, 66%–91%])
				…take MDR TPT with potential mild temporary side effects	96%
				…complete prerequisite steps to determine TPT eligibility:	97%
				Blood test	100%
				Provide sputum sample	
				Obtain chest radiograph	
Study evaluating acceptability of TPT regimen once started					
Purchase et al.^32^	Cape Town, South Africa	Children <5 years of age who were household contacts of a person with MDR-TB disease (n = 27) (median age = 1.9)	Novel child-friendly dispersible 100 mg LFX tablets	Proportion of caregivers that felt…	22/27 (82%)
				– Size of tablet was acceptable	23/25 (92%)
				– Volume of dispersion was acceptable	18/26 (69%)
				– Novel formulation was more palatable than adult formulation	21/26 (81%)
				– Easier to prepare than adult formulation	
Qualitative study assessing caregivers’ experience with administering LFX to children					
Wademan et al.^33^	Cape Town, South Africa	Caregivers and children <5 years of age who were household contacts of persons with MDR-TB disease (from same population as study above) (n = 10 child–caregiver dyads)	Semi-structured interview evaluating experience of administering novel child-friendly LFX formulation	Overall, LFX in children had relatively high acceptability (ability to use medication as directed). Caregivers were willing to administer this formulation to their well children. One of the primary motivators were caregivers’ own experiences with getting sick with MDR-TB and initiating treatment.	
				Caregivers experienced pragmatic difficulties around the financial and care burden on the household due to TPT. Challenges were exacerbated for caregivers who were concurrently on treatment for their own MDR-TB disease, limiting their capacity to care for their children.	
				Participants described novel formulation of LFX as both more palatable, and easier to prepare and administer than the standard of care TPT regimen.	
				Caregivers who received greater social support reported greater capability to ensure adherence to treatment – for themselves and for their children.	

TPT = TB preventive treatment; MDR = multidrug-resistant; HHC = household contact; LFX = levofloxacin; OFX = ofloxacin; MFX = moxifloxacin; ETH = ethionamide; EMB = ethambutol; INH = isoniazid; IQR = interquartile range.

**Table 3.** Summary of studies evaluating the cost-effectiveness of different household contact (HHC) management and TB preventive treatment (TPT) strategies.

Author (year)	Setting	Population	Intervention	Outcome definition/type	Outcome estimates
Fox et al.^34^	USA	Infected HHCs of persons with MDR-TB (n = 1,000) (median age = 37.2 years)	Scenarios compared:	Incident cases of MDR-TB disease averted by TPT per 1,000 contacts	34.4
			1) Status quo – no therapy, clinical review, and chest radiography every 6 months to detect incident disease in first 2 years	Deaths caused by TB averted per 1,000 contacts	3.4
			Full course of 6 months daily, self-administered oral fluoroquinolone therapy,^A^ with monthly clinic visits and routine blood tests	QALYs gained per 1,000 contacts	104
				Health system cost savings per 1,000 contacts (2014 $US)	$5,262,809
Dodd et al.^35^	Global (213 countries)	Estimated number of global MDR-/RR-TB household contacts <15 years of age (n = 227,000, 95% UI: 205,000–252,000)	Scenarios compared:	All incident TB cases reduced by TPT	7,120 (95% UI: 5,800–8,610)
			1) No intervention (i.e., no identification and treatment of contacts, no TPT)	MDR-/RR-TB episodes averted by providing LFX TPT to all children <15 years of age	5,620 (95% UI: 4,540–6,890)
			2) Detection and treatment of TB disease amongst child HHCs, no TPT offered to those without	Deaths averted with detection and treatment of TB disease without TPT	2,350 (95% UI: 1,940–2,790)
			3) Detection and treatment of TB disease + TPT^B^ for different groups of children:	Additional deaths averted by providing LFX TPT to all children <15 years of age	1,240 (95% UI: 970–1,540)^C^
			i) All children aged <5 years, and children <15 years old living with HIV	NNT (TPT with LFX/MFX for all children <15) to prevent one TB episode	38
			ii) All children aged <5 years, and children <15 years old living with HIV or with positive TST	No. of contacts needed to be screened (for TPT in scenario 3a) per death averted	71
			iii) All children aged <15 years	ICER (US$ per DALY) of each scenario relative to no intervention (Scenario 1):	$960
				Scenario 2	$738
				Scenario 3a	$773
				Scenario 3b	$838
				Scenario 3c	171
				Life-years lost, 3% discounted (thousands)	105
				Scenario 1	80.6
				Scenario 2	72.6
				Scenario 3a	70.3
				Scenario 3b	
				Scenario 3c	

MDR-/RR-TB = multidrug-resistant or rifampicin-resistant TB; HHC = household contact; FQ = fluoroquinolone; BDQ = bedaquiline; DLM = delamanid; LFX = levofloxacin; UI = uncertainty interval; ICER = incremental cost-effectiveness ratio; DALY = disease adjusted life-year; NNT = number needed to treat.

A Effectiveness of a 6-month fluoroquinolone regimen was assumed to be 60% (equivalent to 6-month isoniazid treatment for contacts of patients with drug-susceptible TB), due to the lack of evidence from randomised trials.

B For TPT, four different regimens were considered: LFX or MFX (same efficacy but different costs assumed) or BDQ or DLM (also assumed to have same efficacy but different costs).

C Deaths averted varies by TPT regimen and scenario. Further details provided in paper. Generally, BDQ/DLM averted more deaths across all scenarios (240, 320, and 350 for scenarios 1, 2, and 3, respectively).

### Safety of FQ-based TPT among MDR-/RR-TB contacts

Drug-related AEs (of any severity) were reported for 14 of the 16 studies evaluating the safety of FQ-based TPT, but estimates were not pooled due to the heterogeneity in the definitions and severity of AEs (Table 1 and Supplementary Data Figure S1). Four studies reported on any drug-related AEs using FQ monotherapy. One was a study among seven adults reporting no AEs with moxifloxacin (MFX).^17^ The other three studies among child and adolescent household contacts reported rates of mild or moderate AEs of 7%–10% for contacts taking either LFX, MFX, or ofloxacin (OFX).^14,20,21^ No serious or grade 3 or 4 AEs were reported.

In five studies, multidrug FQ-based therapy without PZA was used.^15,16,19,22,25^ Among persons of all ages receiving either LFX or MFX paired with ethambutol (EMB) or ethionamide (ETH), 36 (21%) of 172 contacts from Pakistan reported a grade 1 or 2 AE,^22^ and 56 (54%) of 104 contacts from Micronesia reported AEs of any severity.^15^ In two studies of children and adolescents receiving OFX plus EMB, and high-dose isoniazid in Cape Town, 2 (8%) of 24 children experienced any drug-related AE,^19^ and 6 (3%) of 186 contacts experienced Grade 3 AEs.^25^

Four studies reported on AEs with PZA-containing FQ-based regimens.^13,24,26,28^ In two studies predominantly among child and adolescent contacts in the USA, 18 (82%) of 22 receiving OFX plus PZA^13^ and 26 (100%) of 26 receiving LFX plus PZA^24^ experienced any drug-related AE. In the former study, 3 (14%) of 22 contacts reported serious AEs. The other two studies were among adults receiving LFX plus PZA in Italy and Canada, and reported 9 of 12 (75%) contacts^26^ and 17 of 17 (100%) contacts^28^ experienced any AEs, respectively.

**Figure 2.**
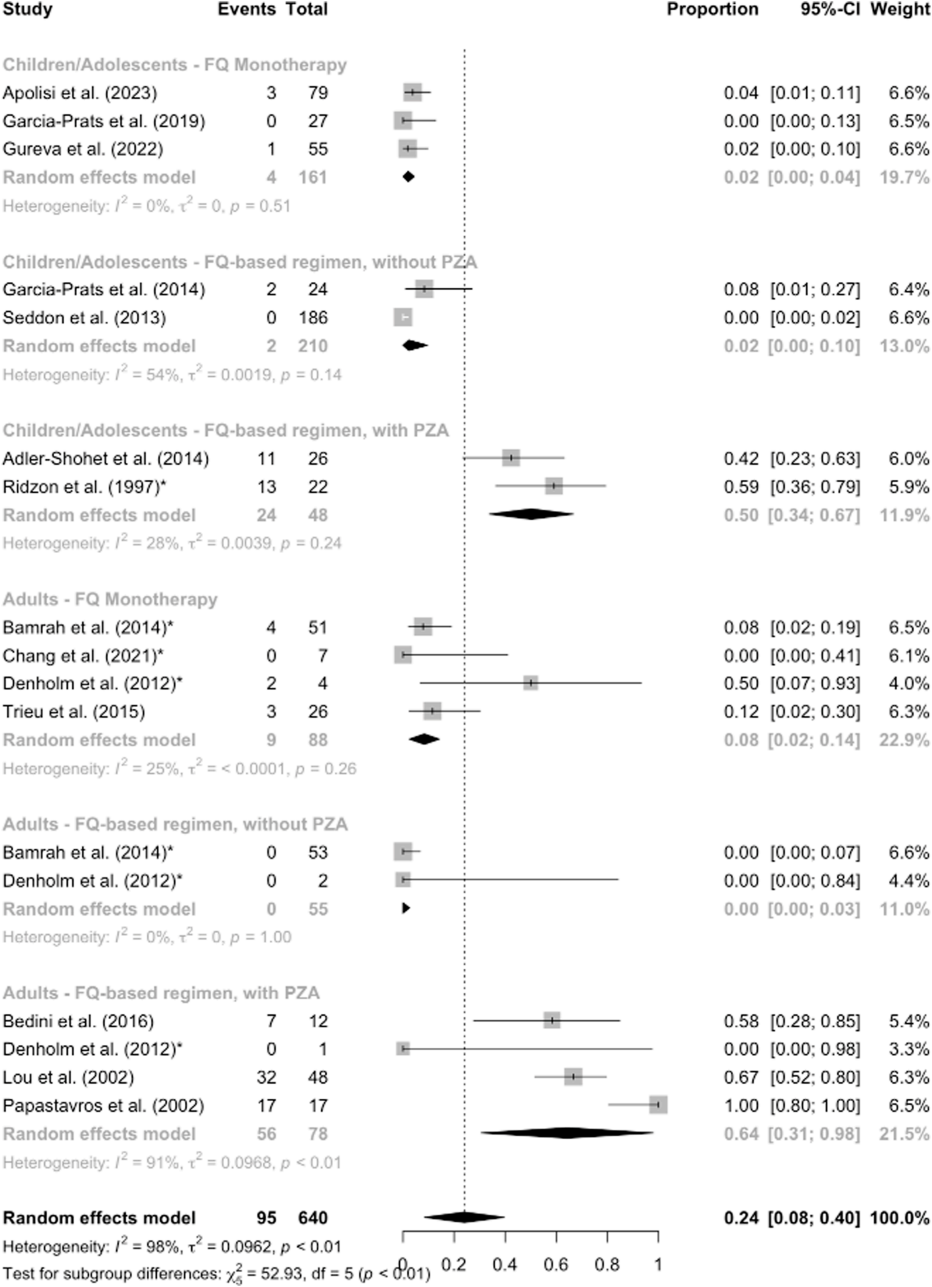
Forest plot of discontinuation of fluoroquinolone (FQ)-based TPT due to drug-related adverse events, stratified by age group, and regimen (random effects model meta-analysis). Note: Populations for some studies (i.e., Denholm et al.^18^) were sub-stratified based on which regimen was taken. Additionally, Ridzon et al.,^24^ Bamrah et al.,^15^ Chang et al.,^17^ and Denholm et al.^18^ included contacts of all ages but were categorised in their respective age groups according to median age of participants. CI = confidence interval; PZA = pyrazinamide.

Drug discontinuation due to AE, reported in 14 studies, was pooled as this outcome was very similar across all studies. Seven studies evaluating FQ monotherapy (with LFX, MFX, OFX, or ciprofloxacin)^14,15,17,18,20,21,29^ had pooled discontinuation rates among children of 2% (95% CI: 0%–4%) and among adults of 8% (95% CI: 2%–14%) (Figure 2). Among the children and adolescents that discontinued monotherapy, all AEs were deemed to be mild or moderate.^14,20,21^ Three discontinued LFX due to gastrointestinal discomfort and one discontinued MFX due to an allergic reaction. One child from the study in Micronesia discontinued MFX monotherapy after simultaneously developing serologically confirmed hepatitis A.^15^ Of the adults who discontinued FQ monotherapy, five discontinued due to nausea or gastrointestinal symptoms, two due to persistent itching or nausea, and one due to muscle and joint pain.^17,18,29^

Among children/adolescents and adults receiving multidrug FQ-based regimens without PZA,^15,19,25^ pooled discontinuation rates were 2% (95% CI: 0%–10%) and 0% (95% CI: 0%–3%) respectively (Figure 2). For studies using PZA-containing FQ regimens,^13,18,24,26–28^ pooled rates were 50% (95% CI: 34%–67%) and 64% (95% CI: 31%–98%) among children and adult contacts, respectively (Figure 2). The most common AE leading to discontinuation was hepatotoxicity.

In a sensitivity analysis excluding small cohorts with ≤10 participants, the pooled estimates of treatment discontinuation did not change, except among adult contacts receiving PZA-containing regimens, where the discontinuation rate increased from 64% (95% CI: 31%–98%) to 77% (95% CI: 51%–100%) (see Supplementary Data Figure S2).

### Acceptability of FQ-based TPT regimens

Three studies reported TPT acceptance among caregivers administering to children,^13,15,21^ and two reported the acceptance of TPT by contacts for themselves.^23,26^ (Table 2). As seen in the supplement (Supplementary Data Figure S3), the pooled proportion of caregivers that accepted that their children would start TPT was 85% (95% CI: 78%–92%) and the pooled proportion of adults that accepted TPT for themselves was 82 (95% CI: 67%–97%).

A qualitative study from eight countries found that 586 (79%) of 743 adult and adolescent contacts were willing to take a hypothetical, newly developed MDR TPT (Table 2).^31^ Knowledge about TB, comfort about speaking with friends and family about potential treatment, and confidence in properly taking MDR TPT were factors positively associated with willingness. Willingness was reduced to 70% if TPT had potential side effects, even if mild and temporary. A subsequent study reported that 278 (93%) of 299 caregivers (aged ≥13 years), of the children or dependants who participated in the same multinational study, were willing to administer daily MDR TPT to their children.^30^ A study conducted in Cape Town found that quantitative measures of acceptability were generally high (69%–92%) among children and caregivers receiving a novel child-friendly LFX formulation (Table 2).^32^ A qualitative study in Cape Town reported that this same formulation had relatively high acceptability among children; caregivers were also willing to administer it to their children (Table 2).^33^ However, respondents had concerns about the financial and care burden, especially caregivers who themselves were on treatment for MDR-TB disease. Caregivers who received greater social support reported greater capability to adhere to MDR disease treatment themselves and ensure MDR TPT adherence for their children.

### Cost-effectiveness of FQ TPT among household contacts exposed to MDR-TB

Fox et al.^34^ estimated that 6 months of daily FQ therapy with monthly clinic visits for household contacts exposed to MDR-TB disease in the USA would result in significant health system cost savings, with 34.4 incident cases of MDR-TB disease and 3.4 deaths averted and 104 quality-adjusted life-years (QALYs) gained per 1,000 contacts (Table 3). Dodd et al.^35^ used global data from 213 countries to evaluate the cost-effectiveness of several contact management strategies among child (<15 years old) household contacts of MDR-TB index patients (Table 3).^34^ The authors estimated that provision of TPT with LFX or MFX added to detection and treatment of co-prevalent TB disease would be more cost-effective than detection and treatment of disease alone. LFX was found to be more cost-effective than delamanid for almost all countries. Targeting TPT to children under five, plus children under 15 with HIV, would avert an additional 870 deaths and 25,000 life-years lost, compared to averting 1,240 deaths and 35,000 life-years lost through provision of TPT to all contacts under 15.

## DISCUSSION

Our review indicates that FQ monotherapy is a safe TPT regimen. No serious AEs were reported, and only 2% of children and adolescents and 8% of adult contacts discontinued therapy due to mild or moderate, or grade 1–2 drug-related AEs. FQ monotherapy had high acceptability among children and adults and was estimated to be more cost-effective than detecting and treating MDR-TB disease among household contacts alone. PZA-containing FQ regimens, on the other hand, were significantly less safe.

Strengths of this review include the evidence from heterogeneous populations across various settings, meaning that the pooled results should be more generalisable to all populations that may benefit from FQ TPT. The review also provides a more comprehensive evaluation of treatment outcomes with FQ monotherapy; these results complement RCT data evaluating 6-month LFX TPT among household contacts in Vietnam and South Africa.^4,5^ However, this review also had several limitations. Since all studies were unblinded and uncontrolled, there may have been bias in selection of those receiving FQ, as well as reporting bias for AEs. Direct comparisons of safety between regimens were not feasible within individual studies, and thus the results were subject to differences in study populations and settings across studies. Clinical heterogeneity for definitions of any drug-related AEs was high, precluding pooled estimates for this outcome. Statistical heterogeneity was high when pooling all studies reporting AEs leading to TPT discontinuation but was acceptable once data were stratified by age and regimen type. Finally, since most studies included relatively small numbers of participants, the outcome estimates in each study were imprecise. However, given the total of 14 studies evaluating each of the safety outcomes, pooled outcome estimates were more precise.

The threshold for considering a certain risk of AEs acceptable is much lower among contacts receiving TPT than among patients treated for MDR-TB disease. We found that FQ monotherapy had the lowest rate of drug-related AEs and a low rate of discontinuation due to AEs, making it the most suitable regimen for MDR-/RR-TB contacts. Pairing FQs with additional drugs (i.e., ETH or EMB) had similarly low treatment discontinuation as FQ monotherapy but had higher rates of any AEs. Given the evidence that FQ monotherapy is efficacious,^4,5^ this regimen seems preferable, given the lower pill burden and complexity for contacts, and lower costs for TB programmes.

Use of PZA was associated with much higher rates of AEs in the studies included in this review, suggesting that PZA-containing FQ regimens should be avoided. This aligns with findings from previous systematic reviews,^7,8^ and is also consistent with experience with the 2RZ regimen. This regimen was initially considered safe and recommended in 2000 by the Centers for Disease Control and Prevention, Infectious Disease Society of America, and American Thoracic Society.^36^ However, this recommendation was retracted in 2003 after 48 reports of severe liver injury including 11 deaths were published.^37^

## CONCLUSION

Published results from two randomised placebo-controlled trials^4,5^ and an accompanying meta-analysis of those trials^6^ indicate that FQ monotherapy is safe and effective. This review provides complementary information from observational and modelling studies in more diverse populations and settings. The findings of the review that FQ monotherapy TPT was safe, and highly acceptable in all settings and populations where this was evaluated, suggest that FQ monotherapy should be acceptable to patients and providers. Given this, plus the findings of health system cost savings, greater aversion of deaths, and reduction in QALYs lost, provision of FQ monotherapy should be a high priority for MDR-/RR-TB contacts.

## Supplementary Material
