# Supplementary material for: Fluoroquinolones for TB preventive treatment of contacts exposed to persons with multidrug-resistant TB: a systematic review and meta-analysis
Source: IJTLD Open. 2026 Jun 15;3(6):364–75. doi: 10.5588/ijtldopen.26.0019 (PMC13268093; doi:10.5588/ijtldopen.26.0019)
Supplement: Supplementary file 1 [file ijtldopen26-0019_supplementarydata1.pdf]

Supplemental Figures

Figure S1. Forest plot of any drug-related adverse events reported among contacts receiving fluoroquinolone-based TPT (excluding cohorts ≤10 participants), stratified by regimen (random effects meta-analysis).

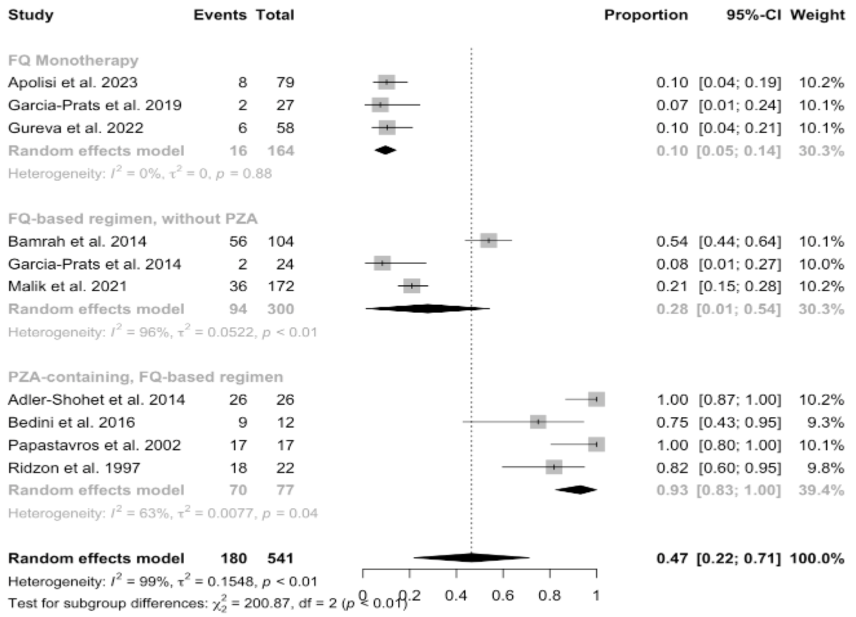

Note: Definitions of drug-related adverse events and their respective severity differed across studies. This forest plot shows the total number of any type of drug-related adverse event reported.

Figure S2. Forest plot with sensitivity analysis (excluding cohorts of ≤ 10 participants) of treatment discontinuation for fluoroquinolone-based TPT due to drug-related adverse events, stratified by age group, and regimen (random effects model meta-analysis).

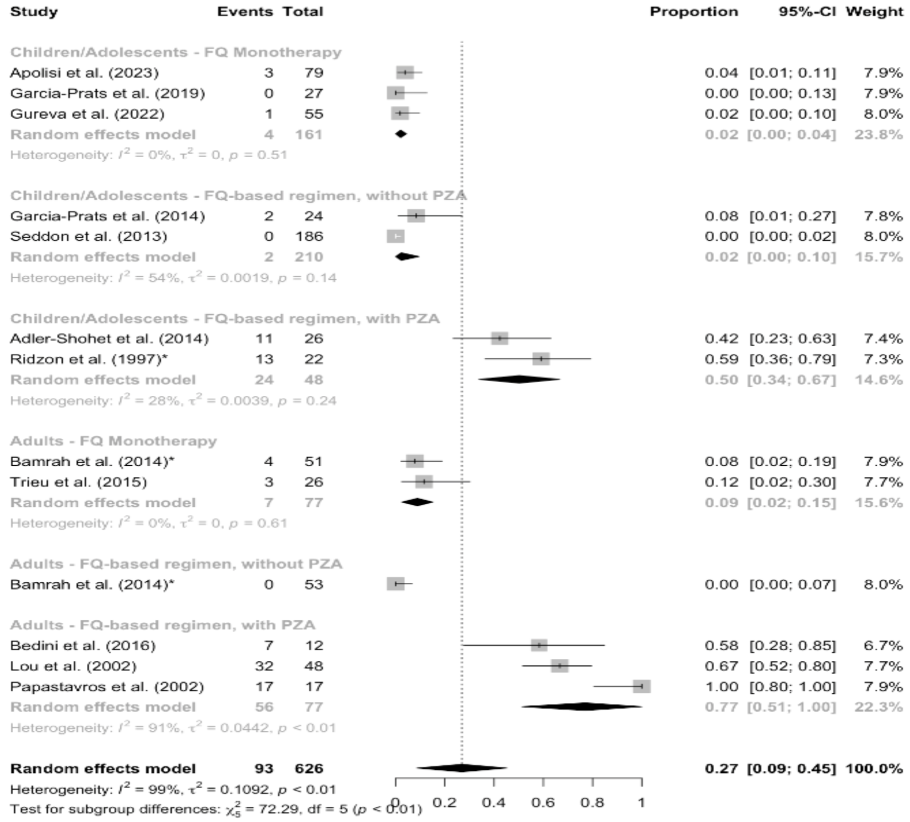

Figure S3. Forest plot of acceptance to start fluoroquinolone-based TPT, stratified by age group (random effects meta-analysis).

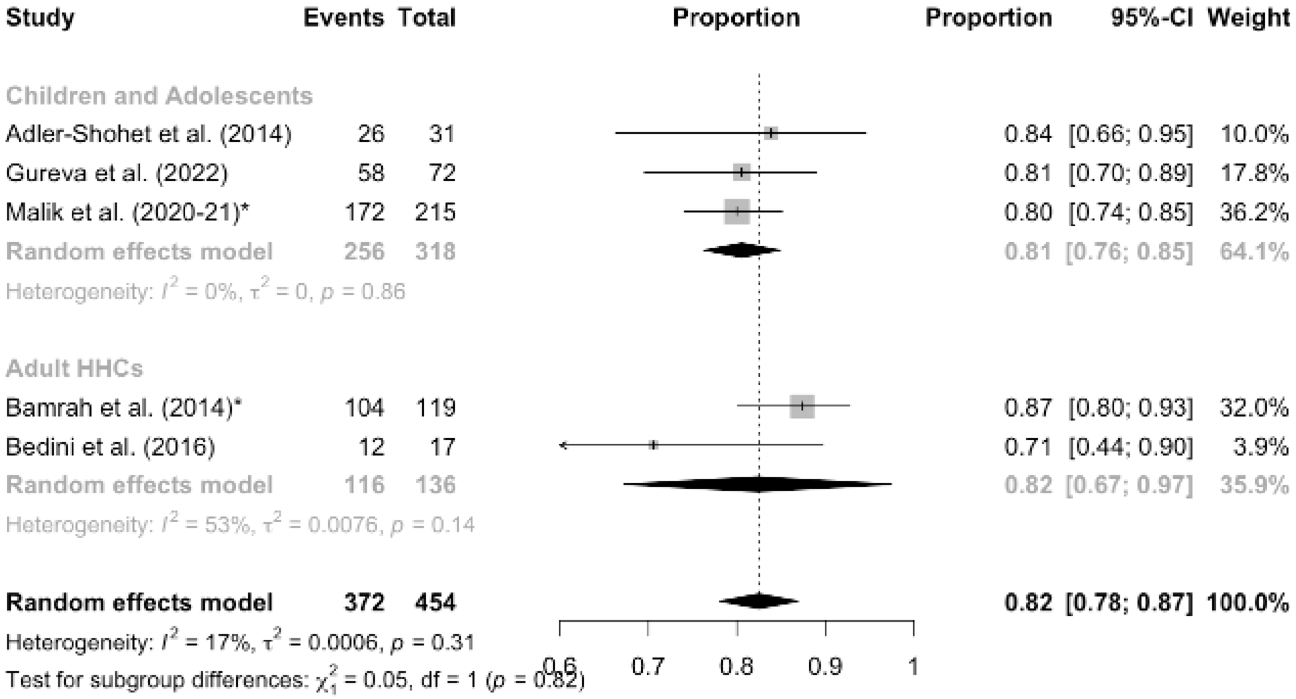

Note: The sub-population of children and adolescents reports on the acceptance of parents or caregivers to start preventive therapy for eligible contacts who were not able to provide consent for themselves. Malik et al. (2020-2021) and Bamrah et al. (2014) included contacts of all ages but were categorized in their respective age groups according to median age of participants.

Supplemental Tables

Table S1. Characteristics of included studies.

First Author, Year	Design/Setting	Population Definition/Description of Contacts	Median Age (years)	Sample size		Regimens	Relevant Outcome(s) Assessed
				Intervention	Control		
Adler-Shohet et al. 2014	Retrospective cohort from an outbreak in a school in California, USA.	Children who spent ≥ 8 cumulative hours in either the same room as the case or the connected classroom.	Mean: 9.6 (range 6-13)	26	5	LFX+PZA for at least 9 months	Drug-related AEs, treatment discontinuation due to drug-related AE, TPT completion ratewe, proportion of parents/caregivers agreeing to start TPT for children
Apolisi. 2023	Prospective cohort study describing an MDR/RR-TB postexposure management program in Khayelitsha, Cape Town, South Africa, 2020-2021.	Household contacts of individuals newly diagnosed with MDR/RR-TB identified from 10 primary care clinics ruled out for TB disease	8.5 (IQR 4.1-11.9)	79	16	Daily 6-month treatment with LFX monotherapy, INH (regular and high dose), DLM (for contacts with FQ-resistant MDR-TB)	Mild or moderate AEs reported, serious AEs, treatment discontinuation due to drug-related AE
Bamrah 2014	Prospective cohort from two outbreaks in Federated States of Micronesia, 2009-2012.	Household contacts defined as persons who had spent at least spent one night in the same household with an infectious MDR-TB patient.	Participant receiving TPT: 24 (range 1-62). Participants not receiving TPT: 32	104	15	MFX only, MFX + EMB, LFX only, LFX + EMB, or LFX +ETH for 12 months	Drug-related AEs, serious AEs, treatment discontinuation due to drug-related AE, TPT completion rate, proportion agreeing to start TPT
Bedini. 2016	Retrospective cohort from an outbreak in a prison in Modena, Italy, 2010-2012.	All individuals who had contact with the index case during the 2 months before the diagnosis.	34 (range 21-51).	12	5	LFX+PZA for 6 months	Drug-related AEs, treatment discontinuation due to drug-related AE, TPT completion rate, proportion agreeing to start TPT
Catho. 2015	Retrospective cohort in Lyon, France, 2007-2012.	Child contacts (<15 years).	6 (range 0.6-14)	1	N/A	PAS/LFX for 6 months	Drug-related AEs
Chang. 2021	Retrospective cohort study at seven chest clinics in New South Wales, Australia, 2000-2016.	Contacts (all ages) of patients diagnosed with MDR-TB	Not reported.	7		MFX for 6 months (12 others received regimens without FQ but are not included for this review)	Drug-related AEs, TPT completion rate
Dodd. 2022	Mathematical modelling study of MDR-/RR-TB household contacts management scenarios for children from 213 countries in 2019 (based on notification data reported to the WHO)	Children younger than 15 years of age who were household contacts of index patients with MDR-/RR-TB from 213 countries	--	--	--	LFX or MFX (same efficacy assumed but different costs), or BDQ or DLM (same efficacy, different costs)	Incident TB cases and deaths averted by LFX TPT, NNT to prevent one TB episode, ICERs and life-years lost given each scenario
Denholm. 2012	Retrospective cohort based on patient records in Victoria, Australia, 1995-2010.	Those with > 8 h of cumulative exposure to potentially infective persons (smear-positive or -negative pulmonary disease).	27 (IQR 17-43).	11	N/A	MFX, RIF/EMB/PZA, CFX/PZA, INH/PZA, EMB/PZA, MFX/EMB, and CFX for 6 or 9 months.	Drug-related AEs, treatment discontinuation due to drug-related AE, TPT completion rate
Fox. 2015	Modelling study (deterministic decision analysis tool) evaluating cost-effectiveness of FQ therapy, USA, 2014	Hypothetical cohort of 1,000 household contacts of patients with MDR-TB who had TB infection, but not TB disease	Assumed median age of 37.2 years	--	--	LFX for 6 months	Incident MDR-TB disease cases and deaths averted per 1,000 contacts, QALYs gained, health system cost savings per 1,000 contacts
Garcia-Prats 2014	Retrospective cohort in the Western Cape Province, South Africa, 2011-2013.	Children aged <15 years with documented exposure to the index case at the day care centre.	2.9 (IQR 2.9-5.2).	24	10	OFX/EMB/high dose INH for 6 months.	Treatment discontinuation due to drug-related AE, TPT completion rate
Garcia-Prats 2019	Pharmacokinetic open-label, lead-in study in Cape Town, South Africa	Children aged <5 years with household contact with an adult MDR pulmonary TB index case diagnosed during the previous 6 months	2.1 (IQR 1.2-2.7)	27	N/A	LFX 100-mg dispersible tablets (observed dose given after overnight fast, samples taken just before and at 1, 2, 4, 6, and 8 hours after dose.	Grade 1/2/3/4 AEs at least possibly related to LFX, treatment discontinuation due to drug-related AE

First Author, Year	Design/Setting	Population Definition/Description of Contacts	Median Age (years)	Sample size		Regimens	Relevant Outcome(s) Assessed
				Intervention	Control		
Gureva. 2022	Prospective cohort study of children identified through a systematic MDR-TB contact investigation in the Arkhangelsk Region, Russian Federation, 2011-2014.	Children aged <18 years consecutively identified as household contacts of confirmed pulmonary MDR-TB cases, with no FQ resistance	7.0 (IQR 4.0-12.3)	58	14	OFX or MFX daily for 9 months	Grade 1 or 2 drug-related AEs, treatment discontinuation due to drug-related AE, TPT completion rate, proportion of parents/caregivers agreeing for child to start TPT
Lou. 2002	Retrospective cohort based on outpatient pharmacy records in University of Pittsburgh Medical Center Health System, USA, 1999	Solid organ transplant recipients with possible exposure to a single index case of MDR-TB	Mean (SD): 51.3 (11.2)	48	N/A	LFX + PZA for 12 months	Treatment discontinuation due to drug-related AE, TPT completion rate
Malik. 2020, 2021	Prospective cohort design with consecutive enrollment of contacts at Indus Hospital in Karachi, Pakistan, 2016-2017.	Household contacts (children and adults) of 100 consecutive index patients beginning treatment for culture-confirmed DR-TB	7 (IQR 3-16)	172	43	LFX+EMB, LFX+ETH, MFX+EMB, MFX+ETH	Grade 1 or 2 drug-related AEs, TPT completion rates, proportion of eligible contacts accepting TPT
Papastavros 2002	Case series design from epidemiological contact investigation of an outbreak in Hamilton, Canada, 2000	Seventeen individuals who had TST reaction ≥ 5 mm (after exposure) and definite contact with at least one of the two index cases	36 (range 18-58)	17	N/A	LFX + PZA	Drug-related AEs, treatment discontinuation due to drug-related AE
Purchase. 2019	Acceptability evaluation study using a caregiver questionnaire in Cape Town, South Africa	Participants taking the novel child-friendly LFX formulation were children <5 years who were household contacts of an adult MDR-TB index case diagnosed during the previous 6 months. The acceptability questionnaire was administered to the child's caregiver to assess drug acceptability.	1.9 (IQR 0.8-2.7)	27	N/A	Novel child friendly LFX dispersible tablet formulation	Quantitative acceptability measures for caregivers of children treated with a novel child friendly LFX formulation (i.e. size of tablet, volume, palatability, ease of preparation)
Ridzon. 1997	Prospective cohort study following school exposure to MDR-TB, California, USA.	High school students and teachers that were exposed to a student with infectious pulmonary MDR-TB	17 (range 15-39)	22	N/A	OFX + PZA daily for 12 months	Drug-related AEs reported, TPT completion rate, treatment discontinuation due to drug-related AEs
Rouzier. 2022	Multi-country, cross-sectional qualitative study (semi-structured KAP questionnaire) conducted in Botswana, Brazil, Haiti, India, Kenya, Peru, South Africa, and Thailand, 2015-2016.	Adult and adolescent household contacts (≥13 years) exposed to MDR-TB who reported caring for children <13 years of age or a dependent of any age.	35 (IQR 27-48)	299	N/A	Hypothetical, daily MDR TPT pill	Willingness of caregivers to administer daily MDR TPT, and to have children complete prerequisite steps to determine TPT eligibility
Seddon. 2013	Prospective cohort study in the Western Cape, South Africa, May 2010 through April 2011	Children <5 years of age or HIV-positive, with significant exposure (defined as living with or having regular daily interaction) to an infectious (sputum smear or culture positive) pulmonary MDR-TB source patient	34 months (IQR 14-47 months)	186	N/A	OFX+EMB+INH (high dose) for 6 months	Grade 1/2/3/4 drug-related AE reported, treatment discontinuation due to drug-related AE.

First Author, Year	Design/Setting	Population Definition/Description of Contacts	Median Age (years)	Sample size Intervention	Sample size Control	Regimens	Relevant Outcome(s) Assessed
Suryavanshi 2019	Multi-country, cross-sectional qualitative study (using KAP questionnaire) conducted in Botswana, Brazil, Haiti, India, Kenya, Peru, South Africa, and Thailand, 2015-2016.	Adult and adolescent household contacts (≥13 years of age) who currently live or have lived in the same dwelling unit or plot of land as an index case, currently share or have shared the same housekeeping arrangements as the index case, and report exposure within 6 months prior to the index case starting MDR-TB treatment.	33 (IQR 22-49)	743	N/A	Hypothetical, newly developed MDR TPT	Willingness of contacts to take hypothetical new TPT with and without potential mild side effects, and to complete prerequisite steps to determine TPT eligibility
Trieu. 2015	Retrospective cohorts from two outbreaks, New York, USA, 2005-2014.	Residents on the floor on which a case-patient resided or visited during the infectious period, and health care staff members who provided direct care to case-patients.	Not reported.	50	N/A	MFX, MFX+PZA (other unspecified regimens were not included in this review)	Treatment discontinuation due to drug-related AE, MFX or MFX+PZA TPT completion rates
Wademan 2022	Exploratory qualitative study on children and caregivers' experiences in Cape Town, South Africa, 2017.	Children <5 years of age who were household contacts of MDR-TB index cases, and their caregivers who administered their novel LFX formulation treatment	Not reported.	10	N/A	Novel child friendly dispersible LFX formulation	Qualitative exploration of caregivers' experience on administering LFX TPT to children

Abbreviations: AE: adverse event; AMC: amoxicillin-clavulanate; AMK: amikacin; BDQ: bedaquiline; CFX: ciprofloxacin; CFZ: clofazimine; DLM: delamanid; DR; drug-resistant; EMB: ethambutol; ETH: ethionamide; FQ: fluoroquinolone; ICER: incremental cost-effectiveness ratio; INH: isoniazid; IQR: interquartile range; KAN: kanamycin; LFX: levofloxacin; LZD: linezolid; MDR-TB: multidrug-resistant tuberculosis; MFX: moxifloxacin; NNT; number needed to treat; OFX: ofloxacin; PAS: para-aminosalicylic acid; PZA: pyrazinamide; RIF: rifampicin; RR-TB: rifampicin-resistant tuberculosis; SD: standard deviation; STR: streptomycin; TPT: TB preventive therapy; TRD: terizidone; TST: tuberculin skin test; QALY: quality-adjusted life years; QTc: corrected QT; XDR: extensively drug-resistant.

Table S2. Risk of bias assessment for included observational studies evaluating safety of fluoroquinolone-based preventive therapy for contacts of persons with MDR TB disease using items from the Newcastle-Ottawa Scale. (Green=Low risk, Yellow=Medium risk, Red=High risk)

Author, Year / Newcastle-Ottawa Scale Item	Adler-Shohet. 2014	Apolisi. 2023	Bamrah. 2014	Bedini. 2016	Catho. 2015	Chang. 2021	Denholm. 2012	Garcia-Prats. 2014	Garcia-Prats. 2019	Gureva. 2022	Lou. 2002	Malik. 2020, 2021	Papastravos. 2002	Ridzon. 1997	Seddon. 2013	Trieu. 2015
Representativeness of exposed cohort (selection bias)	Yellow	Yellow	Yellow	Yellow	Red	Yellow	Yellow	Yellow	Yellow	Green	Yellow	Green	Yellow	Yellow	Green	Yellow
Exposure Ascertainment (selection and information bias)	Green	Yellow	Green	Green	Yellow	Yellow	Yellow	Yellow	Green	Green	Yellow	Green	Yellow	Green	Green	Yellow
Outcome Assessment (information bias, misclassification of outcome)	Green	Green	Green	Yellow	Yellow	Yellow	Red	Yellow	Yellow	Yellow	Yellow	Green	Green	Yellow	Green	Yellow
Adequacy of Follow-Up (information bias)	Green	Yellow	Green	Green	Yellow	Yellow	Yellow	Yellow	Yellow	Red	Yellow	Yellow	Green	Green	Green	Yellow

<u>Table S3</u>. Risk of bias assessment for included cross-sectional studies evaluating acceptability of MDR-TB TPT regimens using items from the AXIS tool.

AXIS Tool for Cross-Sectional Studies	Purchase. 2019	Rouzier. 2022	Suryavanshi. 2019	Wademan. 2022
**Introduction**				
Were the aims/objectives of the study clear?				
**Methods**				
Was the study design appropriate for the stated aims?				
Was the sample size justified?				
Was the target/reference population clearly defined (is it clear who the research was about?)				
Was the sample frame taken from an appropriate population base so that it closely represented the target/reference population under investigation?				
Was the selection process likely to select subjects/participants that were representative of the target/reference population under investigation?				
Were measures undertaken to address and categorize non-responders?				
Were the risk factor and outcome variables measured appropriate to the aims of the study?				
Were the risk factor and outcome variables measured correctly using instruments/measurements that had been trialed, piloted, or published previously?				
Is it clear what was used to determine statistical significance and/or precision estimates? (e.g., p-values, confidence intervals)				
Were the methods (including statistical methods) sufficiently described to enable them to be repeated?				
**Results**				
Were the basic data adequately described?				
Does the response rate raise concerns about non-response bias?				
If appropriate, what information about non-responders was described?				
Were the results internally consistent?				
Were the results presented for all the analyses described in the methods?				
**Discussion**				
Were the authors' discussions and conclusions justified by the results?				
Were the limitations of the study discussed?				
**Other**				
Were there any funding sources or conflicts of interest that may affect the authors' interpretation of the results?				
Was ethical approval or consent of participants attained?				

Low Risk
Medium Risk
High Risk
Not Applicable

Table S4. Risk of bias assessment for included study evaluating cost-effectiveness of MDR-TB TPT regimens using the Joanna Briggs Institute Tool for Economic Evaluation.

**Joanna Briggs Institute Tool for Economic Evaluation**	Dodd. 2022	Fox. 2015
Is there a well-defined question?		
Comprehensive description of alternatives?		
All important and relevant costs and outcomes for each alternative identified?		
Has clinical effectiveness been established?		
Are costs and outcomes measured accurately?		
Are costs and outcomes valued credibly?		
Are costs and outcomes adjusted for differential timing?		
Is there an incremental analysis of costs and consequences?		
Do study results include all issues of concerns to users?		
Are the results generalizable to the setting of interest in the review?		
**Overall Appraisal**		

Low Risk
Medium Risk
High Risk

Supplement: Detailed Search Strategy

PubMed

(Tuberculosis[Mesh] OR tubercul*[tw] OR tubercolosis[tiab] OR TB[ti])

AND

(Tuberculosis, Multidrug-Resistant[MeSH] OR MDR[TW] OR multidrug-resistan*[ALL] OR Multiple-Antibacterial-Drug-Resistance[tiab] OR multi-drug-resistan*[tiab]-OR Rifampicin-Resistant-Tuberculosis[tiab] OR Rifampicin-resistant-TB[tiab] OR Rifampicin-resistan*[tiab] OR RR-TB[tiab] OR XDR-TB[tiab] OR PRE-XDR-TB[tiab] OR extensively-drug-resistant[Tiab] OR drug-resistan*[tiab] OR DR-TB[tiab] OR Rifampin-Resistant-Tuberculosis[tiab] OR Rifampin-resistant-TB[tiab] OR Rifampin-resistan*[tiab] OR ((antibiotic-non-susceptib*[tiab] OR antibiotic-nonsusceptib*[tiab] OR drug-non-susceptib*[tiab] OR drug-nonsusceptib*[tiab] OR antimicrobial-resistan* OR antimicrobial-misuse OR anti-microbial-resistan*) AND multi*[tiab])))

AND

(fluoroquinolone [tiab] OR levofloxacin[tiab] OR moxifloxacin[tiab] OR Antibiotic Prophylaxis[MeSH] OR chemoprevention[MeSH] OR prevent*[ALL] OR prophyla*[ALL] OR "Post-Exposure Prophylaxis"[Mesh] OR "prevention and control" [Subheading] OR "Primary Prevention"[Mesh])

AND

(2016/6/1:2023/9/13[pdat])

TRIP Database

(tb OR tuberculosis OR tubercolosis OR tubercul*)

AND

(drug-resistan* OR antimicrobial-resistan* OR mdr OR multidrug-resistan* OR multi-drug resistan* OR rifampicin-resistan*)

AND

(fluoroquinolone OR levofloxacin OR moxifloxacin OR prevent* OR prophyla* OR latent OR contact OR "secondary prevention")

AND

from_date:2016

Global Health Library (via Ovid)

“multidrug resistant AND tuberculosis”

Limit to yr= “2016-2024”

Embase

1. Exp tuberculosis/
2. "tubercul*".tw.
3. tubercolosis.ab,ti.
4. TB.ti.
5. 1 or 2 or 3 or 4
6. exp multidrug resistant tuberculosis/
7. MDR.tw.
8. "multidrug-resistan*".af.
9. "multi-drug-resistan*".ab,ti.
10. Rifampicin-Resistant-Tuberculosis.ab,ti.
11. Rifampicin-resistant-TB.ab,ti.
12. "Rifampicin-resistan*".ab,ti.
13. RR-TB.ab,ti.
14. XDR-TB.ab,ti.
15. PRE-XDR-TB.ab,ti.
16. Extensively-drug-resistant.ab,ti.
17. "drug-resistan*".ab,ti.
18. DR-TB.ab,ti.
19. Rifampin-Resistant-Tuberculosis.ab,ti.
20. Rifampin-resistant-TB.ab,ti.
21. "Rifampin-resistan*".ab,ti.
22. "antibiotic-non-susceptib*".ab,ti.
23. "antibiotic-nonsusceptib*".ab,ti.
24. "drug-non-susceptib*".ab,ti.
25. "drug-nonsusceptib*".ab,ti.
26. "antimicrobial-resistan*".ab,ti.
27. Antimicrobial-misuse.ab,ti.
28. "multi*".ab,ti.
29. 22 or 23 or 24 or 25 or 26 or 27
30. 28 and 29
31. 6 or 7 or 8 or 9 or 10 or 11 or 12 or 13 or 14 or 15 or 16 or 17 or 18 or 19 or 20 or 21 or 30
32. "fluoroquinolone*".ab,ti.
33. Levofloxacin.ab,ti.
34. Moxifloxacin.ab,ti.
35. Exp 'antibiotic prophylaxis'/
36. Exp chemoprevention/

37. “prevent*”.af.
38. “prophyla*”.af.
39. Exp “Post-Exposure Prophylaxis”/
40. “prevention and control”.sh.
41. Exp “Secondary Prevention”/
42. 32 or 33 or 34 or 35 or 36 or 37 or 38 or 39 or 40 or 41
43. 5 and 31 and 42
44. Limit 43 to dc=20160601-20230913

Cochrane Library (CENTRAL)- Cochrane database of systematic reviews, clinical trials, economic evaluations database

1. (tb):ti,ab,kw
2. tuberculosis:ti,ab,kw
3. MeSH descriptor Tuberculosis explode all trees
4. (#1 OR #2 OR #3)
5. MeSH descriptor Drug Resistance, Multiple, Bacterial explode all trees
6. MDR:ti,ab,kw
7. multidrug-resistan*:ti,ab,kw
8. MeSH descriptor Tuberculosis, Multidrug-Resistant explode all trees
9. (#5 OR #6 OR #7 OR #8)
10. latent:ti,ab,kw
11. contact*:ti,ab,kw
12. MeSH descriptor Contact Tracing explode all trees
13. MeSH descriptor Antibiotic Prophylaxis explode all trees
14. MeSH descriptor Chemoprevention explode all trees
15. prevent*:ti,ab,kw
16. prophyla*:ti,ab,kw
17. (#10 OR #11 OR #12 OR #13 OR #14 OR #15 OR #16)
18. (#4 AND #9 AND #17)

Limit publication date to between June 2016 and October 2023
